# Supplementary material for: Evaluating the Predictive Potential of Patient-Specific Biomechanical Models in Class III Protraction Therapy
Source: Bioengineering (Basel). 2025 Oct 28;12(11):1173. doi: 10.3390/bioengineering12111173 (PMC12649350; doi:10.3390/bioengineering12111173)

Supplementary File S1

Node sets and force distribution per node

Forces were distributed throughout the bone rather than concentrated at a single point. To simulate this in the molar region, 20 nodes were selected on each side of the maxilla: 10 inside and 10 outside the dental arch. This dual-node placement creates realistic force dispersion. Each palatal screw is represented by 7 nodes surrounding the screw's segmentation in the model (Figure 1). Cross-sectional views revealed that node selection extends into the bone due to small holes created during the 1mm mesh construction. The chin-cup pressure is distributed across 43 nodes, matching the actual contact area. For mentoplate cases, each of the four screws is visualized and represented by six nodes (three outside and three inside the mandible) to simulate force dispersion in bone tissue. This method was chosen in an attempt to mimic realistic force distribution in the FEM, rather than creating a generalized model to compare both treatment techniques.

The total force was distributed across all nodes. The table below shows the per-node forces for each of the four patients, with forces varying along the x, y, and z axes due to patient-specific force vector designs. $F_{elastic}$ represents the force applied to the maxilla (upper jaw).

Component	$F_{elastic}$/node	$F_{expansion}$/node	F_{chin}/node
x	0	+/-0.908	0
y	-0.143	0	0.0667
x	-0.133	0	0.0622

Facemask patient 1: overview of forces

Component	$F_{elastic}$/node	$F_{expansion}$/node	F_{chin}/node
x	0	+/-0.908	0
y	-0.141	0	0.071
x	-0.136	0	0.068

Facemask patient 2: overview of forces

Component	$F_{elastic}$/node	$F_{expansion}$/node	F_{chin}/node
x	+/-0.03	+/-0.923	0
y	-0.0724	0	0.1207
x	-0.0589	0	0.0982

Mentoplate patient 1: overview of forces

Component	$F_{elastic}$/node	$F_{expansion}$/node	F_{chin}/node
x	+/-0.043	+/-0.923	0
y	-0.0573	0	0.0955
x	-0.067	0	0.1117

Mentoplate patient 2: overview of forces

Detail of node position in the upper jaw

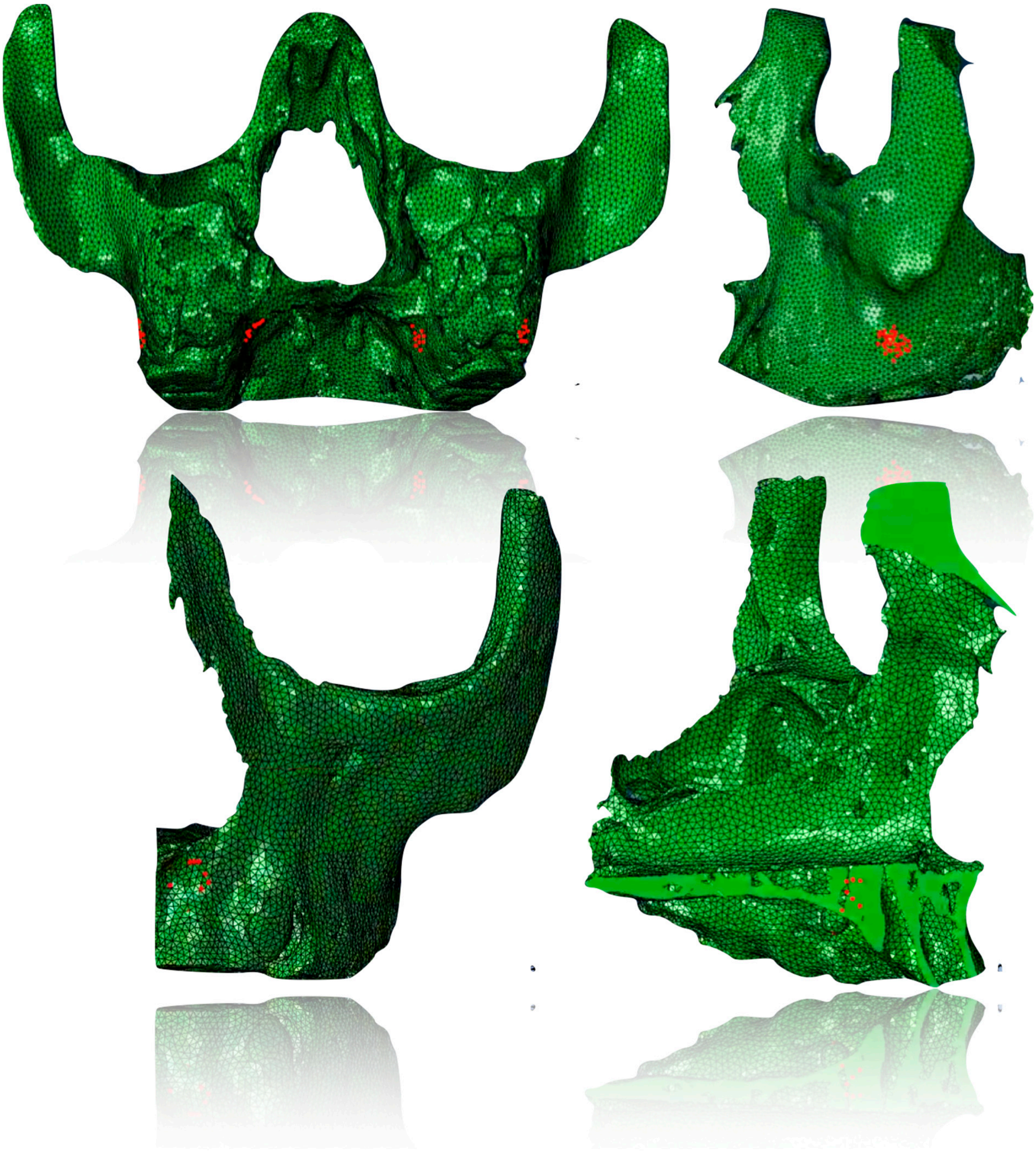

Details of node position in the lower jaw

Upper: mentoplate

Lower: facemask

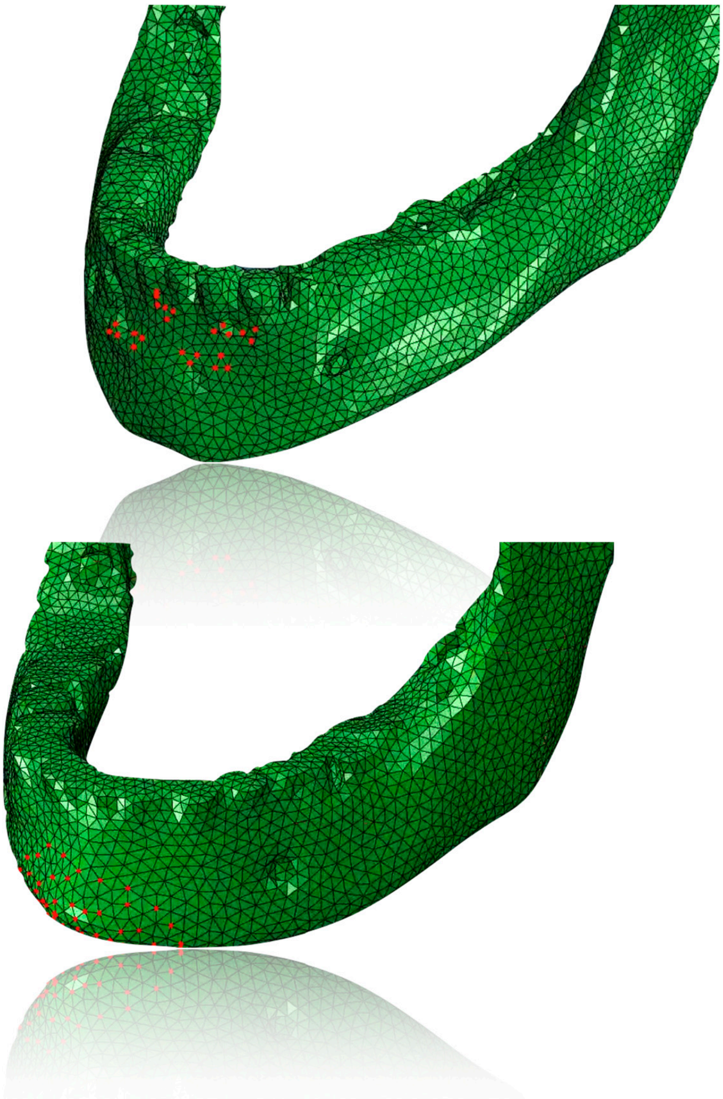

Supplement: Supplementary file 1 [file bioengineering-12-01173-s001.zip › bioengineering-3944941-supplementary.pdf]
